# Urgent Chemotherapy for Aggressive Metastatic Neuroendocrine Cancer With Impending Hepatic Failure: A Case Report

**DOI:** 10.7759/cureus.113596

**Published:** 2026-07-29

**Authors:** Gabriel B Rashba, Lucas Kaplan, David Bolos, Armine Baltayan, Simon Backer

**Affiliations:** 1 Internal Medicine, Olive View - UCLA Medical Center, Sylmar, USA; 2 Hematology and Oncology, Olive View - UCLA Medical Center, Sylmar, USA; 3 Pathology, Olive View - UCLA Medical Center, Sylmar, USA

**Keywords:** benign thyroid nodules, etoposide and carboplatin combination, hepatomegaly, palliative systemic therapy, platinum chemotherapy, poorly differentiated neuroendocrine carcinoma

## Abstract

Neuroendocrine carcinoma is a rare, biologically diverse malignancy that frequently presents with metastatic disease, which can carry a poor prognosis. Its symptomatology is often nonspecific, resulting in delayed diagnosis with substantial tumor burden. Platinum-based chemotherapy remains the cornerstone of treatment, although outcomes remain poor and novel therapeutic strategies are needed. We report a case of newly diagnosed metastatic neuroendocrine cancer in a patient presenting with vague constitutional and gastrointestinal symptoms, ultimately complicated by impending hepatic failure from extensive liver involvement. Initial evaluation raised greater concern for lymphoma or thyroid malignancy, given the pattern of lymphadenopathy and imaging findings. However, tissue biopsy ultimately revealed metastatic neuroendocrine carcinoma. This case underscores the aggressive nature of the poorly differentiated neuroendocrine carcinoma subtype, the importance of early recognition, and the challenges of managing patients with rapidly progressive disease.

## Introduction

Neuroendocrine neoplasms (NENs) comprise a heterogeneous group of epithelial malignancies that are broadly classified into well-differentiated neuroendocrine tumors (NETs) and poorly differentiated neuroendocrine carcinomas (NECs). Poorly differentiated NECs are high-grade malignant neoplasms characterized by neuroendocrine morphology, extensive necrosis, and high mitotic activity [1]. These tumors are distinct from well-differentiated NETs and typically exhibit rapid growth kinetics, early hematogenous dissemination, and poor overall prognosis. Extrapulmonary NECs most commonly arise from the gastrointestinal tract and pancreas, although primary lesions may also originate from other tissues or remain occult despite extensive evaluation [2]. Due to their aggressive biology, most patients with NECs present with advanced, metastatic disease at the time of diagnosis that frequently involves the liver, lymph nodes, or bone [3].

The clinical presentation of metastatic NECs is typically nonspecific. Symptoms may include vague constitutional changes, abdominal pain, weight loss, nausea, or vomiting [1]. Laboratory abnormalities may also be seen that are related to end-organ involvement, such as elevated liver enzymes, hyperbilirubinemia, and electrolyte disturbances. Unlike well-differentiated NETs, poorly differentiated NECs are infrequently associated with hormonally mediated syndromes, which may further delay recognition and diagnosis [4]. Histopathologic evaluation remains essential for diagnosis and typically demonstrates either small-cell or large-cell morphology with positive immunohistochemical staining for neuroendocrine markers such as synaptophysin and chromogranin [5]. In many cases, diagnosis is established only after biopsy of metastatic lesions, particularly hepatic metastases, which are common at presentation. The rapidly progressive nature of these malignancies can lead to fulminant clinical decline, including impending hepatic failure in patients with extensive liver involvement.

Current treatments for metastatic poorly differentiated NECs are largely extrapolated from small-cell lung cancer care, with platinum-based chemotherapy combined with etoposide representing the standard first-line systemic regimen [6]. However, the median overall survival for patients with advanced extrapulmonary NEC remains poor, generally ranging from 10 to 12 months despite treatment [1]. Nevertheless, prompt initiation of systemic therapy is critical, particularly in patients with a high tumor burden or evidence of rapidly progressive organ dysfunction. Urgent chemotherapy may provide meaningful tumor cytoreduction and temporary reversal of life-threatening complications.

Here, we present a case of newly diagnosed, metastatic poorly differentiated NEC presenting with vague constitutional and gastrointestinal symptoms, ultimately diagnosed through biopsy of hepatic lesions and requiring urgent inpatient chemotherapy in the setting of impending hepatic failure.

## Case presentation

A 47-year-old woman with a history of alcohol use disorder, type 2 diabetes, schizoaffective disorder, and obesity presented to the emergency room with three weeks of progressive abdominal pain, nausea, weight loss, and decreased oral intake. The patient reported that her symptoms began shortly after escalating semaglutide therapy from 0.5 mg to 1.0 mg three weeks before presentation, initially attributing her symptoms to a medication side effect. The abdominal pain was persistent, localized primarily to the epigastric region, and unrelated to meals or activity. Associated symptoms included approximately 10-pound unintentional weight loss over 10 days, intermittent dyspnea on exertion, and drenching night sweats for approximately two months. She denied any vomiting, dysphagia, diarrhea, gastrointestinal bleeding, fever, chest pain, dysuria, or skin changes. Social history was notable for prior heavy alcohol use, prolonged incarceration, and residence in a group home for individuals with mental illness. Family history was significant for thyroid malignancy of unknown type in the patient’s mother and maternal grandmother.

In the emergency room, the patient was tachycardic but otherwise afebrile and hemodynamically stable. Physical examination demonstrated a moderately distended abdomen with easily palpable hepatomegaly and tenderness to palpation in the epigastric region and right upper quadrant. No lymphadenopathy was appreciated, and cardiopulmonary examination was unremarkable. Laboratory findings on admission were significant for elevated lactate dehydrogenase (LDH), carcinoembryonic antigen (CEA), white blood cell count, and serum calcium, and prolonged international normalized ratio (INR) and low hemoglobin (Table 1).

**Table 1:** Laboratory values on admission and hospital day 6 WBC: white blood cell; INR: international normalized ratio; LDH: lactate dehydrogenase; CEA: carcinoembryonic antigen; AST: aspartate aminotransferase; ALT: alanine aminotransferase

Test	Results (On Admission)	Results (Hospital Day 6)	Reference Range
WBC	13.8 K/cumm	16.3 K/cumm	4.5-10.0 K/cumm
Hemoglobin	10.0 g/dL	9.1 g/dL	12.0-14.6 g/dL
Platelets	496 K/cumm	598 K/cumm	160-360 K/cumm
INR	2.17	1.42	0.86-1.15
LDH	1,932 U/L	2,374 U/L	98-192 U/L
CEA	57 ng/mL	n/a	<5 ng/mL
Calcium	12.1 mg/dL	9.9 mg/dL	8.9-10.3 mg/dL
Total bilirubin	0.9 mg/dL	3.1 mg/dL	0.1-1.2 mg/dL
AST	104 U/L	227 U/L	15-41 U/L
ALT	37 U/L	70 U/L	14-54 U/L

**Figure 1:**
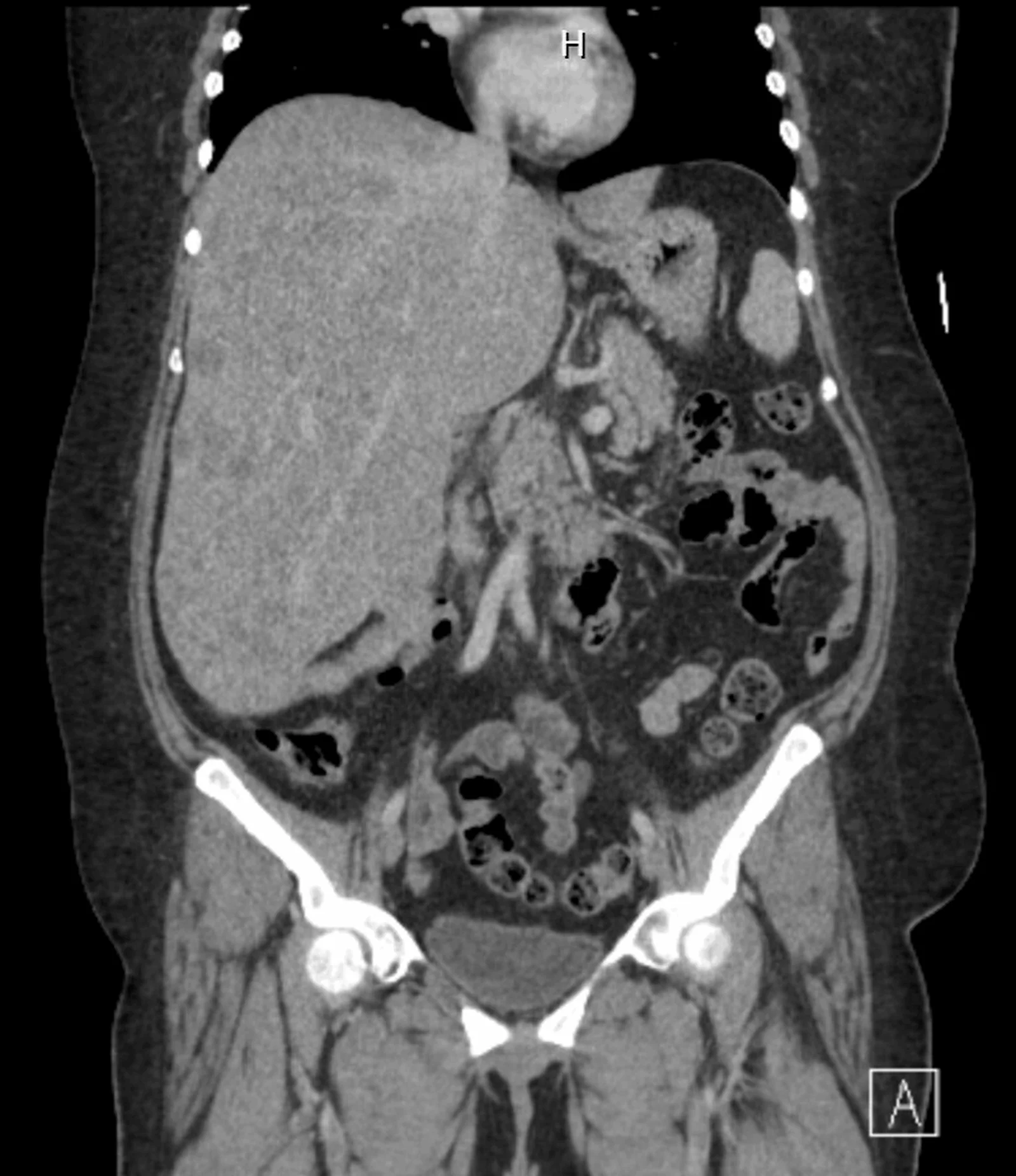
Coronal CT of the abdomen and pelvis demonstrating profound hepatomegaly with multiple mass lesions

A computerized tomography (CT) scan of the abdomen and pelvis demonstrated an enlarged liver with multiple hepatic mass lesions, concerning for metastases (Figure 1).

**Figure 2:**
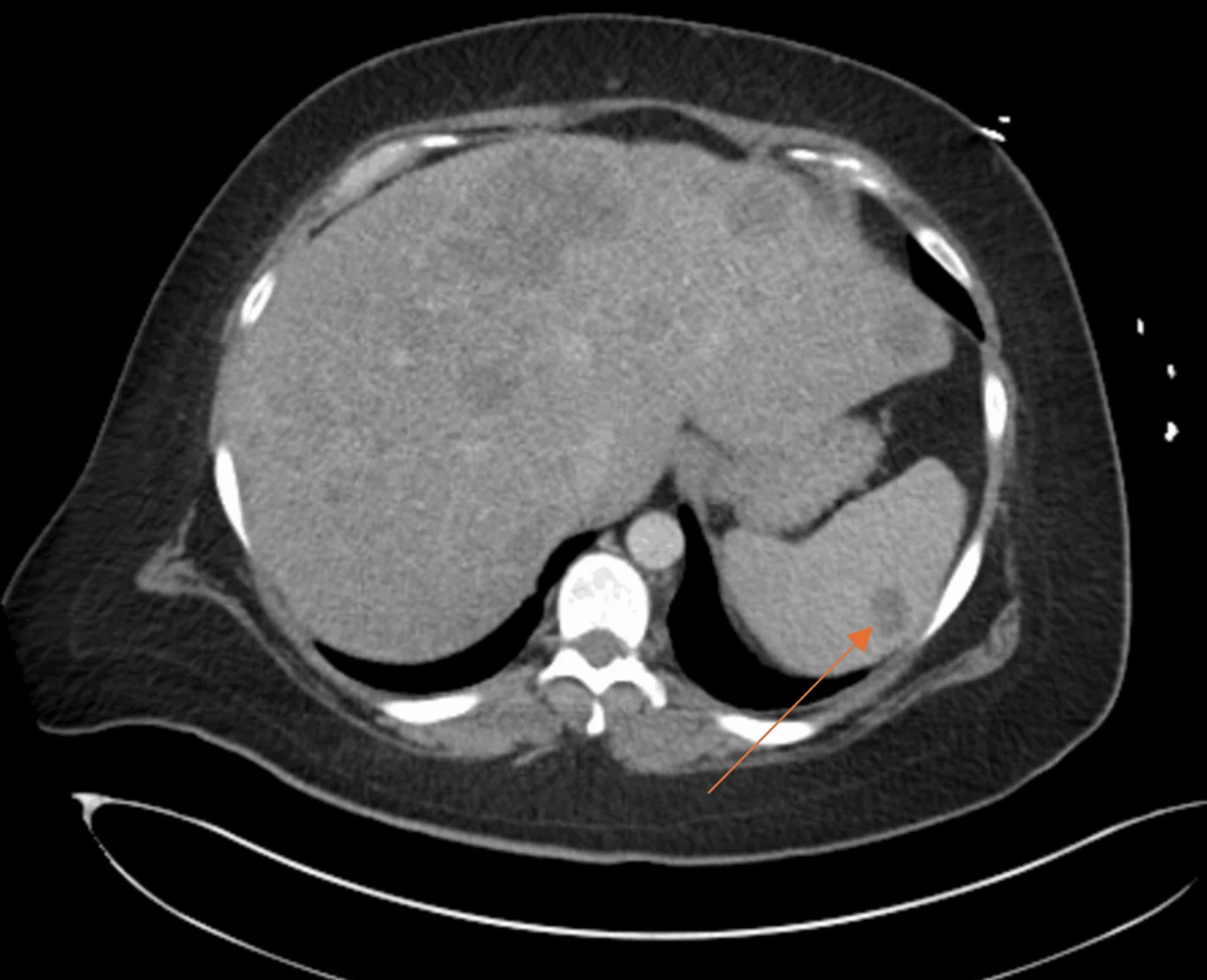
Axial CT of the abdomen and pelvis showing splenic hypodensity (arrow) as well as hepatic mass lesions

Other findings included an indeterminate splenic hypodensity (Figure 2) and a distended gallbladder with gallbladder wall thickening.

**Figure 3:**
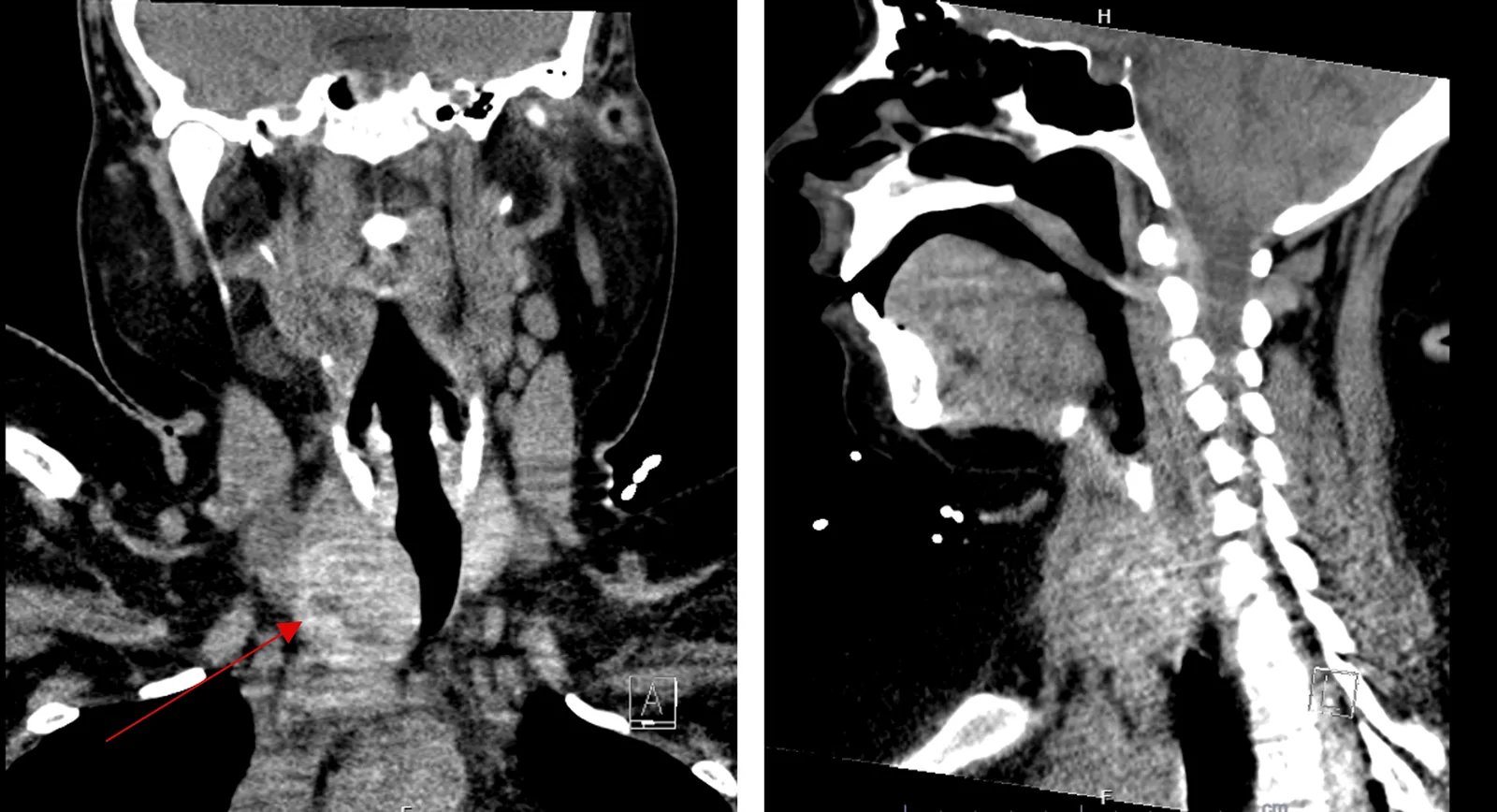
A multiphase CT scan of the neck and parathyroid (coronal, left; sagittal, right) demonstrating an enlarged, irregular right lobe of the thyroid

The patient’s overall presentation was highly concerning for a malignancy. The differential diagnosis was most suspicious for lymphoma or metastatic thyroid cancer based on obtained imaging. Due to the patient’s hypercalcemia and concern for malignancy, the oncology team was consulted, which recommended soft tissue neck and thorax CT for tumor staging. Thyroid ultrasound was also obtained due to the patient’s family history of thyroid malignancy. Neck imaging was significant for a heterogeneous enlarged right thyroid gland, measuring approximately 4.3 x 3.6 x 5.2 cm, with superior mediastinal extension (Figure 3).

**Figure 4:**
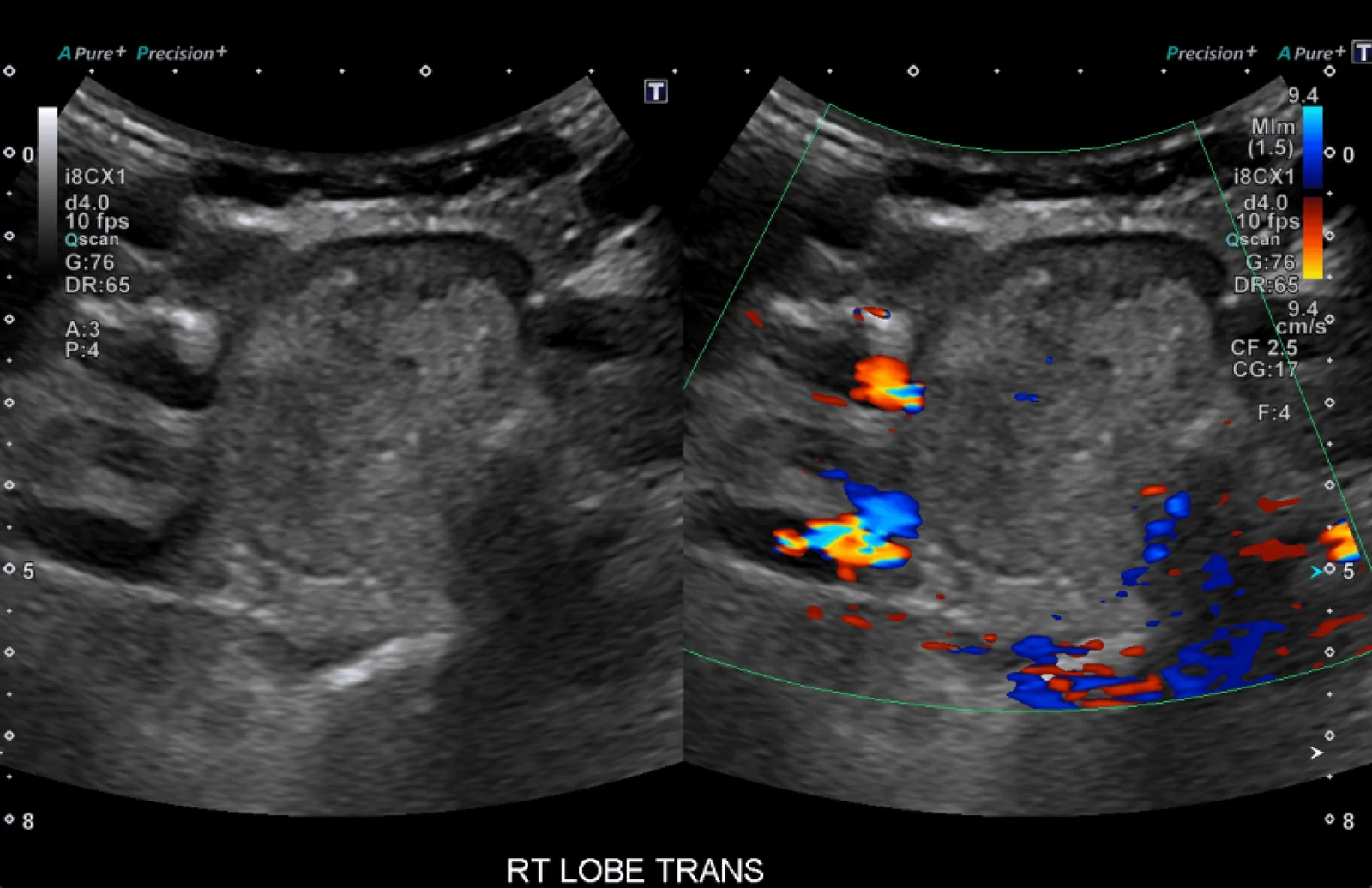
Ultrasound of the thyroid gland demonstrating the right thyroid lobe with an enlarged nodule

There was evidence of mass effect on the adjacent structures, including leftward displacement of the subglottic airway without high-grade stenosis. A thyroid ultrasound was also obtained, confirming the presence of a large right thyroid nodule that occupied most of the right thyroid lobe (Figure 4).

Given her family history of thyroid malignancy and the presence of a large thyroid mass, concerns were also raised for an underlying endocrine neoplasm, particularly because glucagon-like peptide-1 (GLP-1) receptor agonists are contraindicated in patients with a history of medullary thyroid carcinoma or multiple endocrine neoplasia type 2 (MEN2). Interventional radiology was consulted for liver biopsy and fine needle aspiration (FNA) of the thyroid, both of which were obtained on hospital day 3. The patient continued supportive treatment with intravenous (IV) fluids, antiemetic medications, and empiric antibiotics with a gradual improvement in her symptoms. Her hypercalcemia was treated with IV fluids, calcitonin, and a one-time dose of zoledronic acid with improvement in calcium levels.

**Figure 5:**
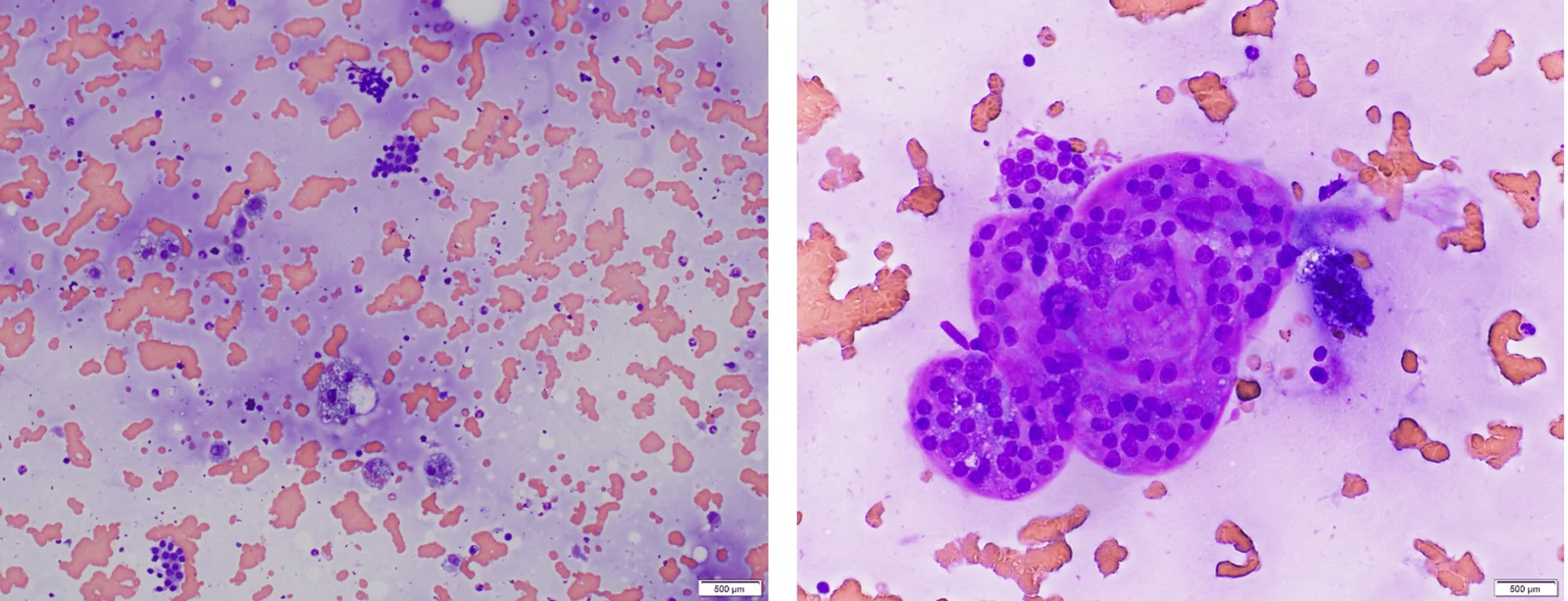
FNA of the thyroid showing histiocytes and benign-appearing follicular cells in a background of loose colloid (left) and a macrofollicle with Hürthle cell changes (right) FNA: fine needle aspiration

On hospital day 6, thyroid FNA results demonstrated a benign follicular nodule without evidence of malignancy (Figure 5).

**Figure 6:**
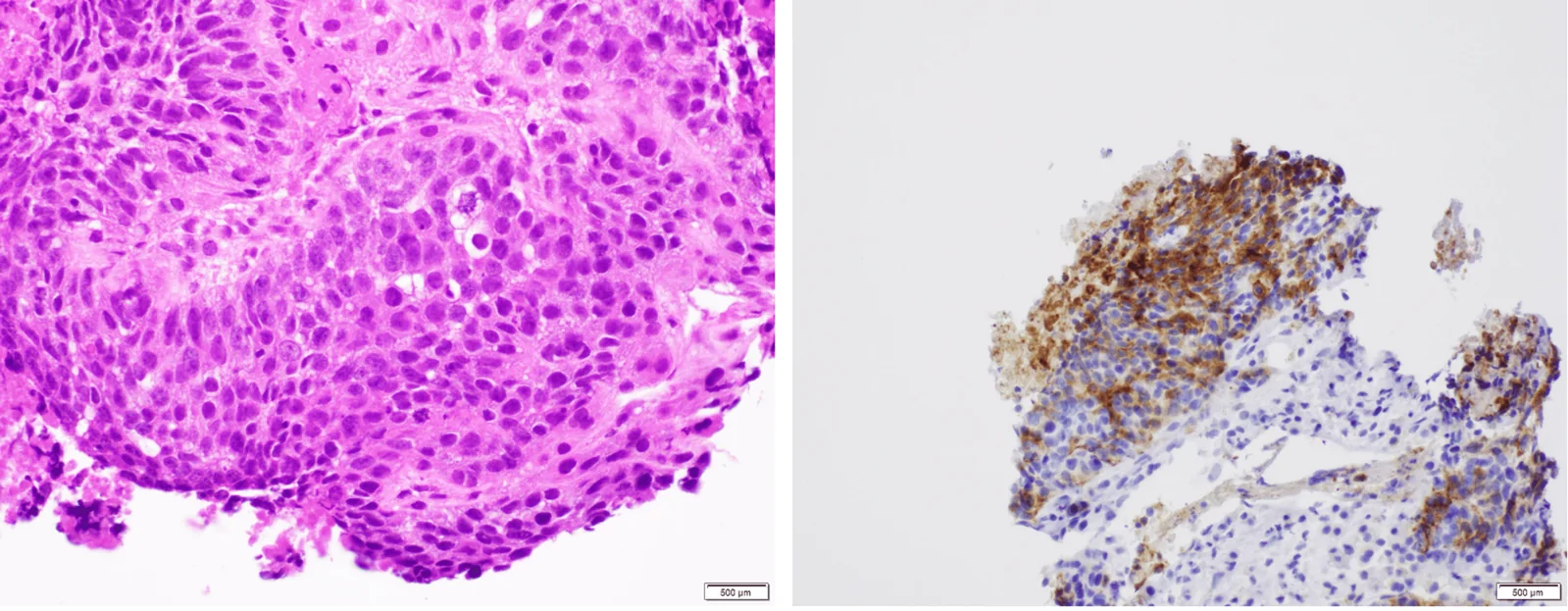
High-power H&E stain of the liver biopsy demonstrating neuroendocrine features (left); IHC stain of the liver biopsy showing positivity for synaptophysin (right) H&E: hematoxylin and eosin; IHC: immunohistochemistry

Liver biopsy revealed poorly differentiated carcinoma with neuroendocrine features and several areas of necrosis, suggestive of a pancreatobiliary primary site of origin. By immunohistochemistry, the tumor was positive for CDX2, CK7, and synaptophysin, while it was negative for CK20, PAX8, GATA3, Hep-par, chromogranin, and p40 (Figure 6).

This diagnosis differed substantially from the initial leading considerations of lymphoma and thyroid malignancy, which immediately altered the management strategy. Rather than pursuing additional diagnostic staging or disease-specific therapies appropriate for lymphoma or thyroid cancer, the diagnosis of poorly differentiated NEC prompted immediate consideration of chemotherapy.

At the time the pathology results became available, the patient’s liver function began to deteriorate with progressively increasing bilirubin and aminotransferase levels (Table 1). Given the risk of impending hepatic failure secondary to extensive hepatic tumor involvement, along with the possibility that continued bilirubin elevation could soon preclude systemic treatment, the decision was made to initiate urgent inpatient chemotherapy. Allopurinol prophylaxis was begun for tumor lysis syndrome, and the patient was hydrated with more aggressive IV fluid resuscitation. The patient received carboplatin (AUC 5) on day 1 and etoposide 60 mg/m² (dose reduced from 100 mg/m² due to hepatic impairment) on days 1-3, with plans for 21-day treatment cycles. Bilirubin levels stabilized at the time of discharge (cycle 1, day 4) with plans for follow-up laboratory checks and further chemotherapy infusions.

The patient ultimately re-presented to the hospital two weeks later with anemia discovered at her nursing facility that was treated with blood transfusions. Repeat imaging at that time showed stable tumor burden, and she was discharged. The patient returned weeks later for cycle 2 of carboplatin and etoposide with no significant acute complications. However, over the course of the next several weeks, the patient was admitted two additional times for ongoing anemia, diffuse pain, and failure to thrive. Several goals-of-care discussions were held with the patient, and her code status was changed to Do Not Resuscitate, Do Not Intubate (DNR/DNI). The oncology team decided to hold further cycles of chemotherapy given the patient’s declining functional status, and she ultimately passed away while hospitalized, only eight weeks after diagnosis with neuroendocrine cancer.

## Discussion

As demonstrated in our patient, poorly differentiated NECs are among the most aggressive neuroendocrine neoplasms and are frequently diagnosed at an advanced stage with extensive metastatic burden. Liver metastases are particularly common at initial presentation and are associated with a significantly worse prognosis and shortened survival [7]. In many large retrospective studies, the median overall survival for metastatic extrapulmonary NEC has been reported to range from approximately 5 to 12 months despite therapy [1,8]. There are even poorer outcomes observed in patients presenting with hepatic dysfunction, elevated LDH, or high Ki-67 proliferative indices [9].

Management of neuroendocrine neoplasms is variable and depends on the primary site, proliferative index, tumor differentiation, and extent of disease. The current limited treatment strategies are summarized in Table 2. Well-differentiated NETs are typically indolent and could be managed with surgical resection, somatostatin analogs, or peptide receptor radionuclide therapy (PRRT) [10]. Somatostatin analogs such as octreotide have demonstrated improved progression-free survival in metastatic well-differentiated NETs, especially in neoplasms that express somatostatin receptors [11]. Everolimus and cabozantinib have also shown some benefit clinically in pancreatic NETs [12,13]. On the other hand, poorly differentiated NECs are generally treated with systemic cytotoxic chemotherapy regardless of the primary tumor origin site. Platinum-based therapy such as carboplatin combined with etoposide remains the most widely accepted first-line treatment regimen [10,14]. Due to its more favorable toxicity profile, carboplatin is often substituted for cisplatin in patients with poor functional status or renal insufficiency [14].

**Table 2:** Summary of current treatment regimens for neuroendocrine tumors and carcinomas NET: neuroendocrine tumor; NEC: neuroendocrine carcinoma; PFS: progression-free survival; OS: overall survival

Author	Year	Malignancy	Treatment	Outcome
Rinke et al. [11]	2009	Metastatic, well-differentiated midgut NETs	Octreotide	Improved PFS
Yao et al. [12]	2011	Pancreatic NETs	Everolimus	Prolonged PFS
Chan et al. [13]	2024	Previously treated advanced pancreatic NETs	Cabozantinib	Improved PFS
Moertel et al. [14]	1991	Poorly differentiated NECs	Etoposide and platinum therapy	Improved OS and PFS

Although treatment for metastatic NECs is often palliative rather than curative, prompt initiation of chemotherapy remains very important due to the high proliferative index of these tumors. Many studies have shown that patients receiving platinum therapy demonstrate improved symptom burden, prolonged survival, and temporary disease stabilization when compared to supportive care alone [14]. Dose-adjusted regimens, such as the reduced-dose etoposide utilized in our patient, may allow treatment delivery even in the setting of hepatic impairment. In the landmark NORDIC NEC study, researchers evaluated 305 patients with advanced gastrointestinal NEC and found a median overall survival of only 11 months among patients receiving palliative chemotherapy, compared with approximately one month in those managed with the best supportive care alone [7]. Findings such as these demonstrate both the lethality of untreated disease and the meaningful survival advantage of systemic therapy.

While initially chemo-sensitive, these carcinomas usually continue to progress, highlighting the need for novel therapeutic agents. Comprehensive molecular profiling has shown that NECs are genetically heterogeneous tumors with frequent changes involving TP53, KRAS, BRAF, APC, and other DNA damage repair pathways [15]. BRAF V600E mutations in particular have been identified in a small subset of high-grade colorectal NECs and could potentially respond to BRAF/MEK inhibition [16]. As sequencing becomes more accessible, routine molecular characterization may help stratify prognosis and personalize therapy in a disease historically managed with empiric chemotherapy alone.

In addition, immunotherapy agents may also present a future therapeutic approach. Single-agent checkpoint inhibitors have shown only modest activity overall in unselected NEC populations, but responses appear more favorable in tumors with high tumor mutational burden (TMB), PD-L1 expression, or MSI-H phenotype [16]. Combination immunotherapy regimens including nivolumab plus ipilimumab have demonstrated more encouraging results in small trials of high-grade neuroendocrine neoplasms [17]. These regimens may allow for a more personalized treatment approach for metastatic NECs.

There are several other emerging therapies that could potentially expand the treatment landscape for patients with advanced NEC in the next several years. Although traditionally used in well-differentiated somatostatin receptor-positive NETs, PRRT is currently being investigated in selected patients with high-grade NECs that are somatostatin receptor positive [18]. Antibody-drug conjugates, bispecific antibodies, and novel cell-surface targets including DLL3 are also under active investigation because of biological similarities between NEC and small-cell lung carcinoma [19]. All these potential future therapies could shift the treatment paradigm away from cytotoxic chemotherapy alone. Nevertheless, our patient’s case highlights the devastating outcomes that can occur despite urgent intervention and underscores the ongoing need for a new therapeutic approach.

## Conclusions

We have presented a case that demonstrates how rapid clinical deterioration can occur when extensive hepatic involvement is present in metastatic neuroendocrine cancer. In our patient, urgent inpatient administration of carboplatin and dose-adjusted etoposide was pursued with the intent of achieving rapid tumor cytoreduction and preserving hepatic function in the setting of worsening liver dysfunction. Platinum-based chemotherapy remains the current first-line treatment and may provide meaningful symptom relief, but outcomes continue to remain very poor. Although the patient experienced only transient stabilization of bilirubin before ultimately developing progressive disease and dying shortly after diagnosis, her clinical course highlights both the limited effectiveness of current treatment strategies and the urgent need for more effective therapies. This case also highlights the importance of maintaining a broad differential diagnosis in patients presenting with nonspecific constitutional symptoms and metastatic disease of unclear origin, as poorly differentiated neuroendocrine carcinoma may mimic more common malignancies such as lymphoma. Emerging strategies, including comprehensive molecular profiling, immunotherapy, PRRT, and other targeted approaches, hold promise for improving future outcomes. Until then, early recognition and prompt initiation of systemic therapy remain critical in patients with metastatic NEC, particularly when organ-threatening complications such as impending hepatic failure are present.
